# Glycerol monolaurate ameliorates DSS-induced acute colitis by inhibiting infiltration of Th17, neutrophils, macrophages and altering the gut microbiota

**DOI:** 10.3389/fnut.2022.911315

**Published:** 2022-08-12

**Authors:** Ke-Jie He, Jia-Hui Dong, Xiao-Mei Ouyang, Ya-Ni Huo, Xiao-Shen Cheng, Ying Lin, Yue Li, Guoyu Gong, Jingjing Liu, Jian-Lin Ren, Bayasi Guleng

**Affiliations:** ^1^Department of Gastroenterology, Zhongshan Hospital of Xiamen University, School of Medicine, Xiamen University, Xiamen, China; ^2^Binhai County People's Hospital, Yancheng, China; ^3^Guangdong Institute of Gastroenterology, The Sixth Affiliated Hospital of Sun Yat-sen University, Guangzhou, China; ^4^Cancer Research Center and Institute of Microbial Ecology, School of Medicine, Xiamen University, Xiamen, China

**Keywords:** glycerol monolaurate, colitis, immunomodulation, gut microbiota, MAPK, NF-κB

## Abstract

**Background and aims:**

Inflammatory bowel disease (IBD) places a heavy medical burden on countries and families due to repeated and prolonged attacks, and the incidence and prevalence of IBD are increasing worldwide. Therefore, finding an effective treatment is a matter of great urgency. Glycerol monolaurate (GML), which has a twelve-carbon chain, is a compound naturally found in human breast milk. Some studies have shown that GML has antibacterial and anti-inflammatory effects. However, the specific mechanism of action remains unclear.

**Methods:**

Acute colitis was established in mice using 3% DSS, and glycerol monolaurate (500 mg·kg−^1^) was administered for two weeks. QPCR and western blotting were performed to examine the inflammatory status. Mice described were subjected to flow cytometry analysis for immune cell activation.

**Results:**

GML treated alleviated macroscopic symptoms such as shortened colons, increased spleen weight, and caused weight loss in mice with DSS-induced colitis. In addition, GML decreased the expression of pro-inflammatory factors (NF-α, IL-1β and IL-1α) and increased the expression of anti-inflammatory factors (IL-10 and TGF-β). GML inhibited the activation of the MAPK and NF-κB signalling pathways, improved tissue damage, and increased the expression of intestinal tight junction proteins. In addition, LPMCs extracted from intestinal tissue via flow cytometry showed that GML treatment led to a decrease of Th17 cells, Neutrophils and Macrophages. 16S rDNA sequencing showed that GML increased the abundance of commensal bacterium such as Akkermansia and Lactobacillus murinus.

**Conclusions:**

We showed that oral administration of GML ameliorated DSS-induced colitis by inhibiting infiltration of Th17 cells, Neutrophils, and Macrophages, protecting the intestinal mucosal barrier and altered the abundance of commensal bacterium. This study provides new insights into the biological function and therapeutic potential of GML in the treatment of IBD.

## Introduction

Inflammatory bowel disease (IBD) is an autoimmune disorder that includes Crohn's disease and ulcerative colitis. The clinical manifestations of IBD cause suffering in patients and include abdominal pain, diarrhoea and mucous-containing bloody stools (1). In addition, the global incidence and prevalence of IBD are on the rise (2). The recurrence of IBD outbreaks and the inability of current medical treatments to completely cure IBD place a great medical burden on national economies and family finances (3). Some studies have indicated that IBD can disturb the intestinal microecology and the intestinal barrier and immune balance. Therefore, it is closely related to immunity, gut microbes, genes and the environment (4–6). The current treatment methods mainly involve the use of aminosalicylic acid, glucocorticoids, immune regulators, antibiotics, and biological agents (7). The primary goals of using drugs to treat IBD are to induce remission and to prevent recurrence (8). Although these drugs relief symptoms of the patients, they cause intolerable adverse effects on the other hands. (9). Therefore, understanding the pathogenesis of IBD and identifying effective treatment methods are of great importance.

Due to the restricted safety and reliability of current clinical drugs for long-term use in the treatment of patients with IBD, it is necessary to find a drug that not only alleviates the disease status but also has fewer side effects (10). Glycerol monolaurate (GML), which has a twelve-carbon chain, is a compound naturally found in human breast milk and is also a medium-chain fatty acid that is naturally found in plant products, such as coconut oil (11–14). GML is often used as an antiseptic (15, 16) and anti-inflammatory agent in foods and cosmetics (17, 18).

As a medium-chain fatty acid, GML not only provides energy and regulates the immunologic balance (19–21) but also kills harmful pathogens such as Salmonella (22), enterotoxic *Escherichia coli* (23), and *Staphylococcus aureus* (11, 24–28). Interestingly, GML had no effect on probiotics such as Lactobacillus (29–31). Some studies have reported that removing the natural ingredient GML from breast milk greatly reduces the effectiveness of breast milk against harmful pathogens (11). In addition, GML is also effective in the treatment of some patients with viral infection (12, 32, 33). Recent studies have reported that GML can reduce suspected cases of COVID-19 and reduce the C-reactive protein level in patients (33). In addition, GML treatment could reduce virus infection and transmission. Recent studies have reported that GML treatment could reduce transmission of COVID-19 and reduce the C-reactive protein level in patients (33). A report by Li Q et al. showed that application of a GML gel in HIV-infected macaques could effectively prevent the spread of HIV and reduce the immune response of the human body to a certain extent (34). In addition, administering of GML to mice with obesity induced by a high-fat diet could reduce body weight (BW), exhibiting effective lowering of blood glucose and lipid levels, reducing the levels of inflammatory cytokines such as TNF-α, IL-6 in mice and increasing the expression of ZO-1, Occludin and Claudin-1, which could protect the intestinal mucosal barrier very well (35, 36).

With the continuous development of science and technology, the understanding of and attention given to intestinal microecology are increasing, and this is considered to be an indispensable and important factor associated with the occurrence and progression of IBD (10). However, because of the complexity and diversity of the interactions among the intestinal barrier, immunity and gut microbes, much more research on this subject is required. There is some evidence indicating that GML also plays a positive role in maintaining the intestinal microecological balance. GML can increase the abundance of commensal bacterium such as Akkermansia muciniphila, which has a good effect on DSS-induced colitis and colorectal cancer in mice and can increase the production of short-chain fatty acids, such as propionic acid and butyric acid (35–38).

Although GML has a promising future in combating inflammation, inhibiting harmful pathogens, and regulating the intestinal microecology, the mechanisms involved remain poorly explored. We found that GML could decrease the CD4+ T and Th17 cells in lamina propria mononuclear cells (LPMCs). Our results indicated that GML could ameliorates DSS-induced acute colitis by inhibiting infiltration of Th17, neutrophils, macrophages and altering the gut microbiota.

## Materials and methods

### Mice

Male specific pathogen-free (SPF) C57BL/6 mice (6–8 weeks old, 20–25 g in weight) were purchased from Xiamen University Laboratory Animal Center (Xiamen, Fujian) and raised in the SPF Animal Room of Xiamen University Laboratory Animal Center. The animals were kept at a temperature of 20–24°C and humidity of 50 ± 5%. All animal experiments were conducted in accordance with the regulations of Xiamen University Laboratory Animal Center. All mice were adapted for a week before starting the study. Fifty mice were randomly divided into three groups: the Con group (*n* = 10), DSS group (*n* = 20) and DSS+GML group (*n* = 20). All animal experiments were approved by the Animal Research Committee of Xiamen University.

### Induction and treatment of experimental colitis

Studies have shown that higher GML treatment (450 mg kg-1) ameliorates HFD-induced metabolic disorders, supported by prevented visceral fat deposition, improved hyperlipidemia, modulated hepatic lipid metabolism, and reduced serum proinflammatory cytokine, TNF-α. Additionally, all doses of GML attenuated circulating lipopolysaccharide load and insulin resistance (36). So we chose the concentration of 250 mg kg-1, 500 mg kg-1, 1,000 mg kg-1 gavage. The concentration is converted to a specific mass of 5 mgGML, 10 mgGML, 20 mgGML. The male mice were treated with 3.0% (w/v) DSS (molecular mass 36,000–50,000 Da; MP Biomedical, LLCz, Santa Ana, CA) *via* drinking water for 5 days in the DSS and DSS +GML groups, and on day 5, the water was removed and replaced with normal water. Normal mice were treated with normal water. Oral GML was started 7 days prior to the DSS once every two days and continued until the end of the DSS. All groups of mice were gavaged at same time points. GML was purchased from Aladdin Reagent Co., LTD (shanghai, China). Mice in the Con group received sterile water at the same dose. In addition, all mice were weighed daily (at 15:00). After the model was completed, the mice were killed, samples were collected, intestinal tissue was collected for length measurement, spleens were weighed, and mouse sera and faeces were collected for subsequent analysis.

### Pathological histology analysis

After the mice were sacrificed, the colon length was measured, and the distal colon was washed with phosphate-buffered saline (PBS, pH = 7.3), rolled into swiss curly hair with a toothpick, and embedded in paraffin. The samples were cut into 6-μm-thick sections and stained with haematoxylin-eosin (HE) (ServiceBio, Wuhan, China). Finally, the colon morphology was observed by using light microscopy (Motic VM1). The colonic tissue was scored according to the pathological scoring criteria (39).

### RT-qPCR

After the mice were sacrificed, total mRNA from intestinal tissue was extracted from the same part of the colon with TRIzol. Then, cDNA was synthesized using the Reverse Transcription Kit (with dsDNase) (Biosharp, Hefei, China). Quantitative PCR was performed using SYBR Green qPCR Mix (BioSharp, Hefei, China). The level of mRNA was normalized to the GAPDH expression level, and the results were analysed using the 2–ΔΔCt method. The primers were designed and synthesized by Sangon Biotechnology (Shanghai) Co., Ltd (Supplementary Table 1).

### ELISA

The levels of the cytokines TGF-β and IL-1β were measured in serum using enzyme-linked immunosorbent assay (ELISA) kits (Jiangsu Meimian Industrial Co., Ltd., Jiangsu, China) according to the manufacturer's instructions, and the absorbance at a wavelength of 450 nm was measured using a microplate analyser.

### Western blotting

Colon tissues were added to RIPA (SolarBio, Beijing, China) lysate containing 1% protease and 1% phosphatase inhibitors (SolarBio, Beijing, China). Protease and phosphatase inhibit degradation of sample proteins. For Western Blot analysis, 20 μg of protein of colon tissues were separated by SDS-polyacrylamide gel electrophoresis (4% stacking gel, 12% running gel). The concentration of primary antibody was determined to be 1:2000. Then, the samples were ground into homogenate using a tissue grinder. The homogenate was centrifuged at 14,000 rpm and 4°C for 15 min to collect the supernatant. Protein concentration was measured using the BCA Protein Assay Kit (SolarBio, Beijing, China) according to the manufacturer's instructions. The samples (20 μg/lane) were placed in a 4–15% gradient gel and electrophoresed at a constant voltage to isolate proteins, which were transferred to nitrocellulose membranes. Then, the membranes were blocked with 5% skim milk and incubated overnight with the primary antibody at 4°C, followed by incubation with an HRP-conjugated secondary antibody. Finally, the protein bands were detected using an ECL test kit (Signalway Antibody, CA). Primary antibodies against ERK1/2 (# 9102S), P-ERK1/2 (#4370), NF-κB (# 8242S p65), P-NF-κB (#3031, p-p65), and GAPDH (# 5174S), were purchased from Cell Signaling Technology.

### IHC

Immunochemistry (IHC) was performed using the OCT-embedded sample. OCT sections were frozen at −20°C in OCT and cryostat sectioned (20 μm). Non-specific staining blockers were avoided with the IHC background blocker. The concentration of primary antibody was determined to be 1:2000. The samples were cut into 6-μm-thick slices and then mounted on glass slides coated with poly(L-lysine) and dried. First, endogenous peroxidase was blocked, and nonspecific staining blockers were added. Then, primary antibody was added, and the sample was incubated overnight at 4°C. A biotin-labelled secondary antibody was added the next day, and then, a Streptomyces anti-biotin protein (MXB Biotechnologies, Fuzhou, China) was added after incubation at room temperature for approximately 10 min. Finally, DAB chromogenic solution (MXB Biotechnologies, Fuzhou, China) was used for colour development, which was visualized using a bioptic biological microscope (Motic VM1). Primary antibodies against ZO-1 (RRID: AB 2757321) and Occludin (RRID: AB 2764486) (ABclonal Technology, Wuhan, China) were used.

### Cytometric bead arrays of serum cytokines

Antibody-conjugated microspheres were prepared (BD Pharmingen, N.J.) and mixed in the appropriate proportion, and then, 50 μL each of serum and PE Detection Reagent were added, and the mixture was incubated at room temperature away from light for 3 h. Due to the low concentration of inflammatory factors in the serum, the serum is not diluted. The mixture was washed and centrifuged to discard the supernatant. Finally, the microspheres were resuspended in 300 μL of washing solution and then detected by flow cytometry (FCM; Beckman Coulter). Microsphere calibration of the instrument was required before using the machine. Finally, the results were analysed with FCAP Array software.

### FCM analysis

Spleen and colorectal tissue were collected from the mice. The spleen was ground with the flat bottom of a syringe piston to homogenize the splenocytes. RBCs were lysed by using the 10X RBC lysing solution (SolarBio, Beijing, China). The supernatant was discarded, and the red blood cell lysate was resuspended to dissolve red blood cells. The spleen tissue was carefully ground and filtered through a cellular filter with a diameter of 100 μm, and a single-cell suspension was prepared. Red blood cells were removed with erythrocyte lysate and then stained with Fixable Stain 510 (FVS510; BD Bioscience, N.J.) and blocked with Fc-block for approximately 30 min, followed by staining of CD45 (30-F11, BD Bioscience, N.J.), CD11B (M1/70, BD Bioscience, N.J.), LY-6G (1A8, BD Bioscience, N.J.). Finally, the samples were detected by FCM. The intestinal tissue of the mice was washed with PBS, cut into 1 cm fragments and placed in predigestion solution, which was configured using D-Hanks solution, including ethylenediaminetetraacetic acid (EDTA 1 mg/mL) and dithiothreitol (DTT 0.15 mg/mL), for approximately 20 min. After predigestion, the intestinal tissue was cut up and added to digestive juices, containing collagenase and DNase, at 37°C for 1 h and 30 min. Then, the digested tissue was filtered and centrifuged to obtain a single-cell suspension, which was passed through Percoll for collecting LPMCs. Then, the cells were stained with F4/80(FVS510; BD Bioscience, N.J.), Fixable Stain 510 (FVS510; BD Bioscience, N.J.) and blocked with Fc-block for approximately 30 min. Next, CD45 (30-F11, BD Bioscience, N.J.), CD3 (145-2C11, BD Bioscience, N.J.), CD4 (RM4-5, BD Bioscience, N.J.) were stained. Finally, the cells were stimulated with PMA, ionomycin, and brefeldin and then stained for IL-17 (TC11-18H10, BD Bioscience, N.J.) and detected by FCM. The concentration of antibody and Fc-block (BD Bioscience, N.J.) was determined to be 1:100. 500,000 cells were collected using flow cytometry (FCM; Beckman Coulter). Percoll (4, 8%), collagenase (1:200) and DNAse (1:200) were purchased from (SolarBio, Beijing, China). 500000 cells were stimulated for intracellular cytokine staining. The stimulants (1:100) and brefeldin (1:100) were purchased from BD Bioscience, N.J. Cells were stimulated for 6 h. After washing and centrifugation, a membrane-breaking fixative solution (BD Biosciences) was used according to the manufacturer's instructions. Cells were fixed 15 min for intracellular cytokine staining.

### Analysis of the gut microbiota

DNA was extracted from the faeces of mice using HiPure Soil DNA Kit B (Guangzhou Magen Biotechnology Co., Ltd). The DNA concentration was measured using the Equalbit dsDNA HS Assay Kit. Then, PCR amplification and library construction were carried out using 20–330 ng of DNA as a template. In addition, PCR primers were used to amplify two highly variable regions, namely, the V3 and V4 regions (40), on the 16S rDNA of prokaryotes. Then, an index sequence was added to the end of the 16S rDNA PCR product for subsequent sequencing. After the library was purified by magnetic beads, the concentration of the library was determined by enzyme labelling, and the fragment size was detected by agarose gel electrophoresis (41). PE250/FE300 paired-end sequencing was performed according to the instructions for Illumina MiSeq/NovaSeq (Illumina, San Diego, CA, USA). After mass filtering, chimaeric sequences were removed, and the sequences obtained were used for OTU clustering. VSEARCH (1.9.6) was used for sequence clustering (sequence similarity was set to 97%). Based on the results of the OTU analysis, the species abundance and diversity of the community were reflected by α diversity indices such as Shannon and Chao1 by random extraction of the sample sequences. (Un)weighted UniFrac analysis was used to determine whether there were significant microbial community differences among the samples. The principal component analysis (PCA), principal coordinate analysis (PCOA) and non-metric multidimensional scaping (NMDS) results were visualized as maps of β-diversity, and ANOSIM for intergroup difference analysis was conducted to determine whether the grouping was meaningful by comparing the intergroup and intragroup differences in rank values. Metastats is an analytical method that uses rigorous statistical methods identify species that show differences in abundance between two microbiomes. LEfSe compares the different species among multiple groups and shows the microbial community or species structure with the differences among groups at each level in the evolutionary branching tree diagram. In addition, PICRUST pathway prediction was performed based on the KEGG pathway database to identify functional genes from the microbial communities by reconstructing unseen states (42). Magnetic bead purification (Beckman Coulter, Brea, CA, USA) followed by the PCR, normalization and pooling were performed according to Illumina's 16S metagenomic sequencing library preparation protocol (Illumina Ltd., San Diego, CA, USA). Amplicons were generated, cleaned, indexed, and sequenced according to the Illumina-demonstrated 16S metagenomic sequencing library preparation protocol with certain modifications. Bacterial 16S ribosomal DNA (rDNA) sequence amplification and MiSeq sequencing of the V3–V4 region of 16S rDNA was subjected to high-throughput sequencing analysis. Nextera XT Index Kit (Illumina inc.) was used for the preparation of the amplicon library as per the 16S metagenomic sequencing (Illumina inc.). 16S sequence processing Reads retrieved from 16S amplicon sequencing was analyzed using the LotuS (1.62) pipeline62. The 16S sequencing data were quality-trimmed using Sickle (https://github.com/najoshi/sickle). The reference library used was the Curated MicroSEQ(R) 16S Reference Library v2013.1; sequencing data was analyzed using the QIIME 1.8.0 software package (Caporaso et al.). Further analysis of 16S sequencing was done on R statistical computing platform with the use of the Vegan package (https://cran.r-project.org/web/packages/vegan/vegan.pdf).

### Statistical analysis

The data are expressed as the mean ± standard error of the mean. GraphPad Prism 8.0 software (CA, United States) was used for visualization and analysis. Student's *t*-test or one-way analysis of variance (ANOVA), followed by the Tukey test for multiple comparisons, was performed to determine significance. A *P-*value < 0.05 was considered significant.

## Results

### GML alleviates the DSS-induced colitis *in vivo*

**Figure 1:**
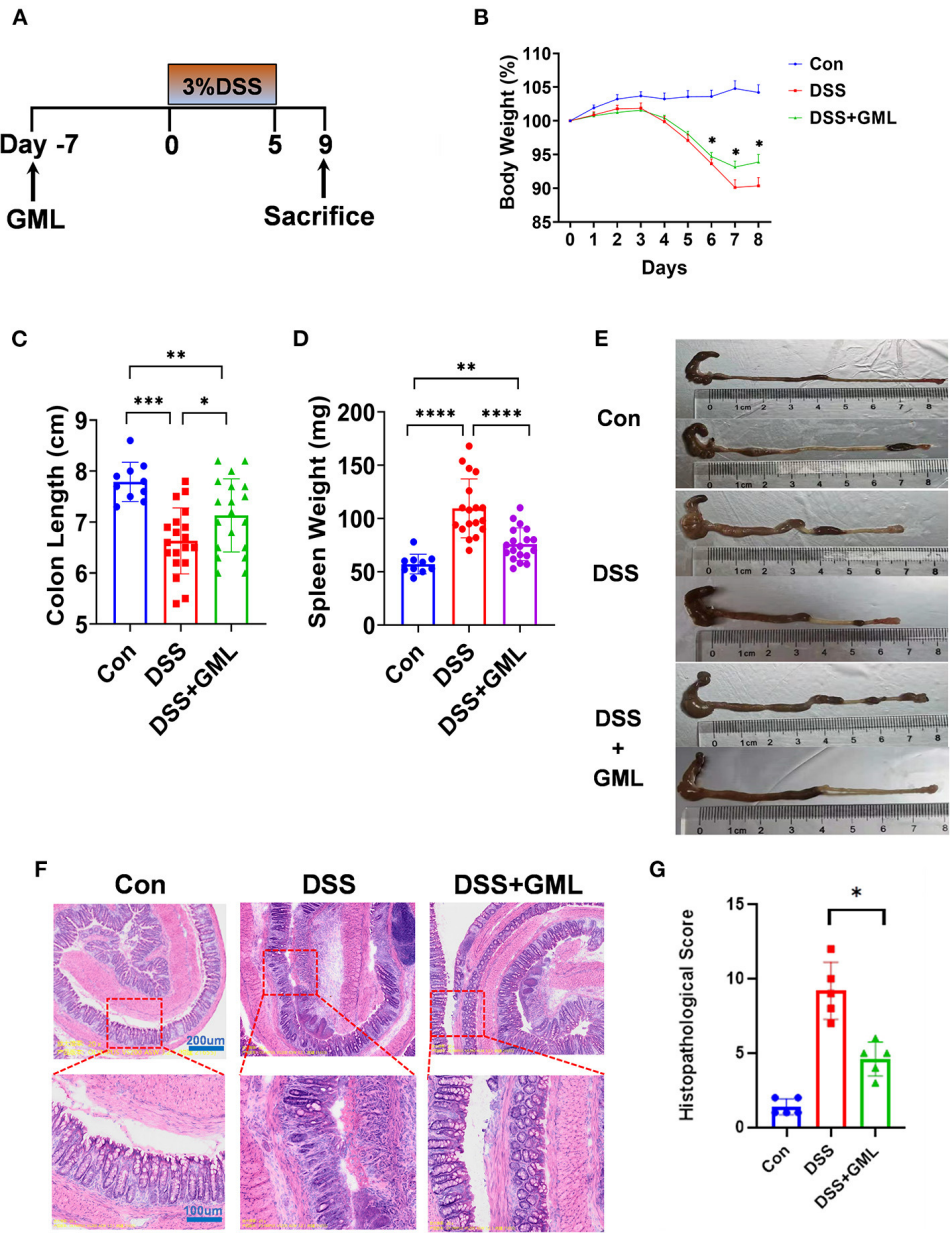
Oral administration of GML alleviated DSS-induced acute colitis symptoms in mice. **(A)** Experimental design and arrangement. **(B)** The body weight of the mice was assessed throughout the experiment and is expressed as a percentage change relative to the initial body weight before DSS administration. **(C,E)** The colon length was measured. **(D)** The spleen weight was measured. **(F)** The distal colon was stained with HE. **(G)** Histopathological score. The data are expressed as the means ± SEMs (Con group *n* = 10, DSS group *n* = 20, DSS+GML group *n* = 20). *P < 0.05; **P < 0.01; ***P < 0.001; ****P < 0.0001.

Experimental colitis induced by DSS is a classic model for the study of the pathogenesis of IBD and the development of treatment techniques and drugs for IBD. The experimental design is shown in the Figure 1A. Before the experiment began, the concentration gradient of the GML was explored (Supplementary Figures S1A,B). Severe acute colitis in mice was induced by oral administration of 3% DSS for 5 days. The results showed that the percentage change in BW of the DSS+GML group began to recover faster from day 6 than that of the DSS group (Figure 1B). Compared with the spleen weight of the Con group, the spleen weight of the DSS group increased sharply, while the spleen weight of the GML group decreased (Figure 1D). The colon length of the DSS group was significantly shortened compared with the DSS+GML group (Figures 1C,E). These results suggest that GML has a certain alleviation effect on DSS-induced colitis.

To further study the protective effect of GML on DSS-induced colitis, colon tissues of mice were collected and sliced for HE staining, as shown in figure (Figures 1F,G). The con group section showed a normal physiological structure, an intact mucosa, and no inflammatory infiltration. Compared with the con group, the mice treated with DSS showed more severe acute colitis, and the mucosal sites were disorganized and unclear, with obvious inflammatory infiltrates, ulcers and oedema. However, the mice treated with GML exhibited reduced pathological damage due to DSS-induced colitis. The lesions were characterized by reduced inflammatory cell infiltration and slight epithelial ulceration. The structure was more complete than that of the DSS group.

### GML regulated the expression of cytokines in colon tissue

**Figure 2:**
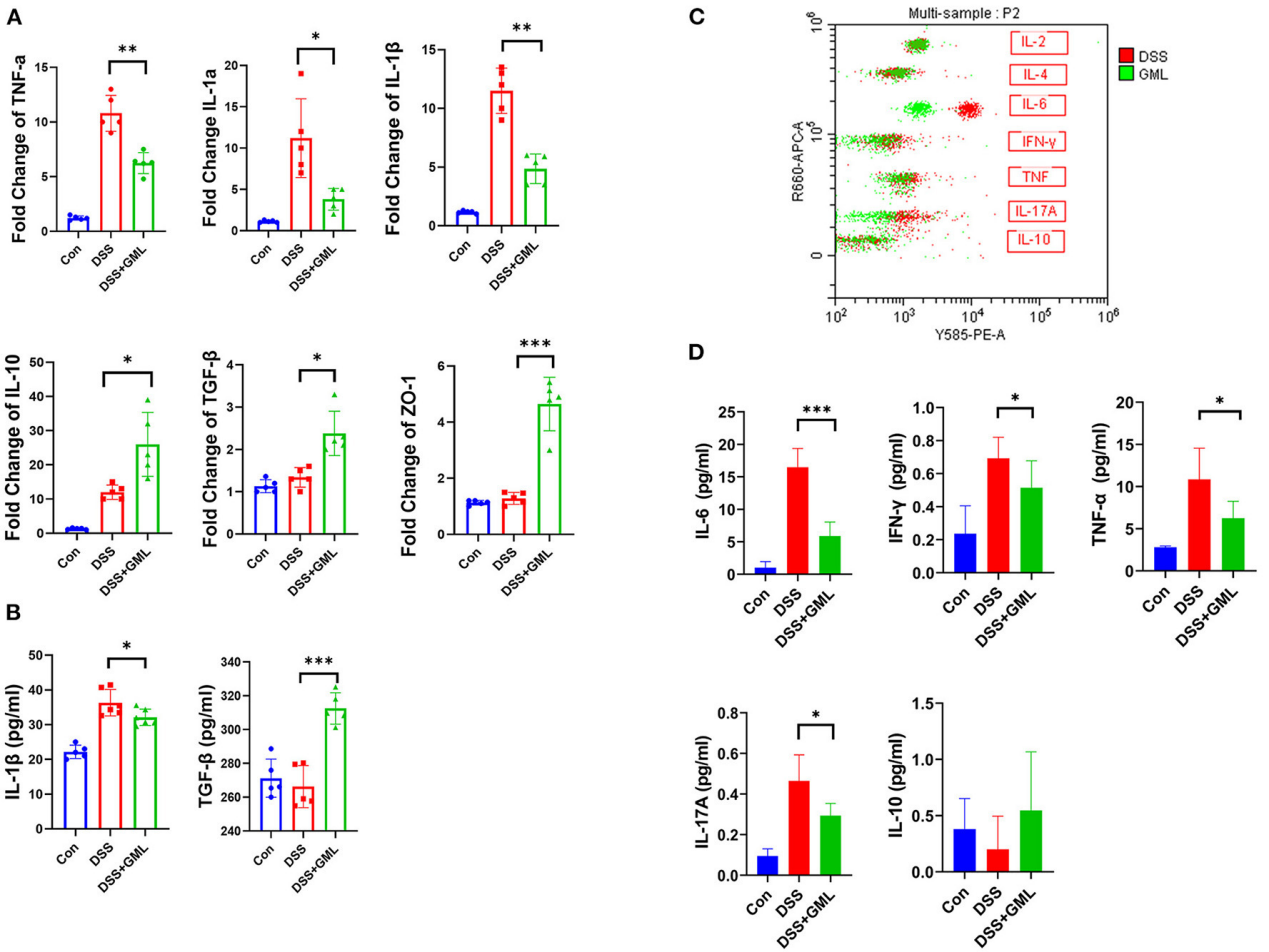
GML decreased the expression of pro-inflammatory cytokines and increased the expression of anti-inflammatory cytokines in DSS-challenged mice. **(A)** mRNA expression levels of TNF-α, IL-1β, IL-1α, TGF-β, IL-10, ZO-1. **(B)** Concentrations of IL-1β and TGF-β in serum by ELISA. **(C)** Results of cytometric bead array immunoassay analysis. **(D)** Quantitative analysis of inflammatory factors (IFN-γ, TNF-α, IL-6, IL-17A and IL-10) in serum. The data are expressed as the means ± SDs (*n* = 5). *P < 0.05; **P < 0.01; ***P < 0.001.

Cytokines play an important role in the occurrence and development of intestinal inflammation, so to further research the effect of GML on colitis, we extracted total RNA from intestinal tissue and performed RT-qPCR. The fold change indicates the change of mRNA according to real-time qPCR. As shown in Figure 2, the TNF-α, IL-1β and IL-1α levels were significantly increased in the DSS group but significantly decreased in the DSS+GML group. The IL-10 and TGF-β levels were significantly decreased in the DSS group but increased in the DSS+GML group (Figure 2A). The results showed that GML could effectively inhibit the mRNA expression of pro-inflammatory factors and increase the mRNA expression of anti-inflammatory factors. Moreover, ELISA results showed that the level of the inflammatory cytokine IL-1β was decreased after GML treatment compared with that in the DSS group, while the TGF-β level was significantly increased (Figure 2B). In addition, the expression of ZO-1 was increased in the GML group compared with the DSS group (Figure 2A), indicating that GML could protect the intestinal mucosal barrier to some extent. Compared with those in the Con group, the levels of the pro-inflammatory factors IFN-γ, TNF-α and IL-6 were significantly increased in the DSS group. However, this change was reversed by the application of GML; the levels of the inflammatory factors were significantly decreased, and IL-17A also showed a tendency to decrease (Figures 2C,D). The standard curve is in the supplemental data (Supplementary Figure S1C). The results showed that GML could effectively inhibit the expression of inflammatory factors.

### GML treatment inhibited the NF-κB and MAPK signalling pathways and attenuated DSS-induced damage to tight junctions in the colon tissues

**Figure 3:**
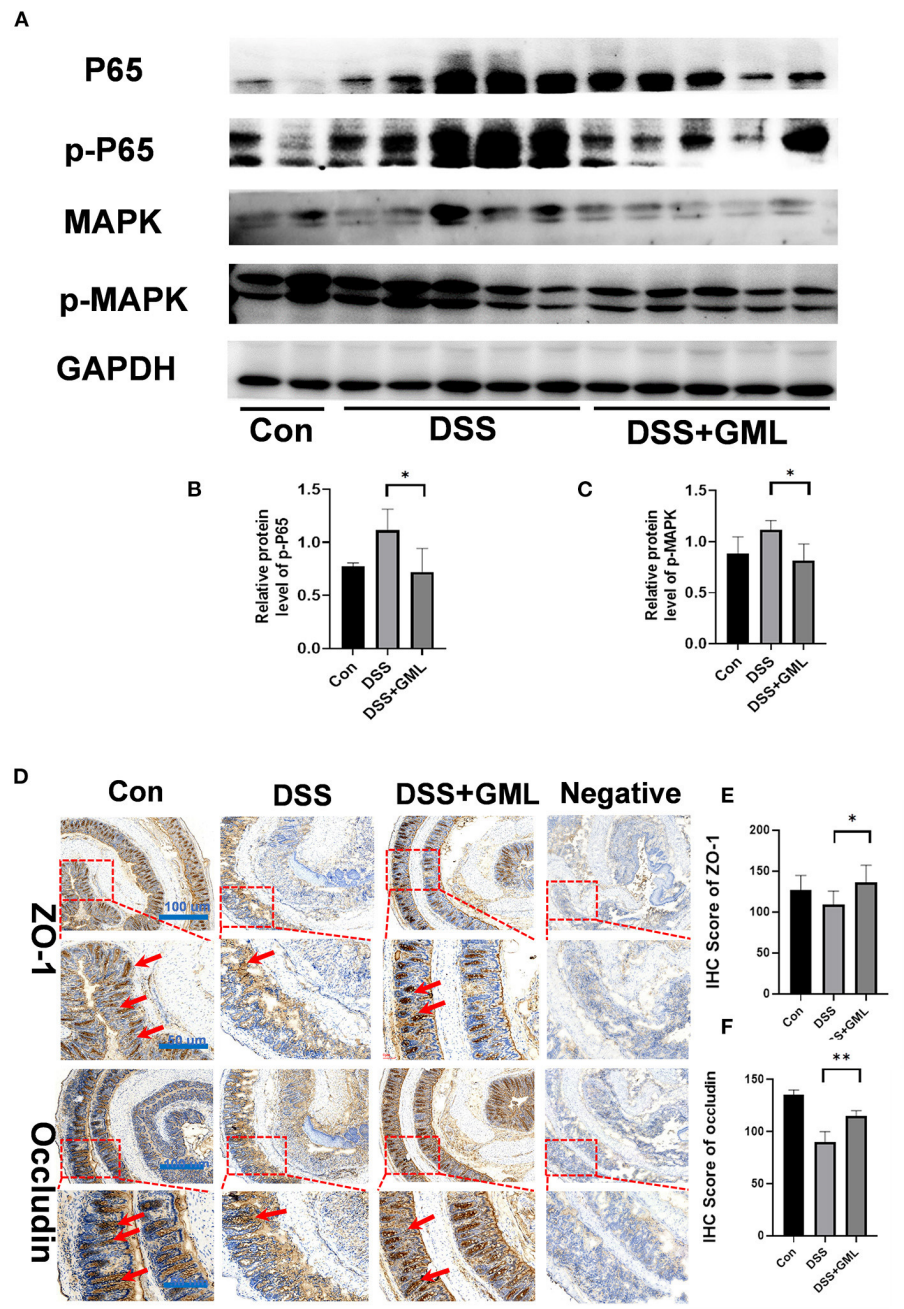
GML treatment inhibited the NF-κB and MAPK signalling pathways and attenuated DSS-induced damage to tight junctions in the colon tissues. **(A)** Representative protein bands for p-p65, p65, p-MAPK, and MAPK. **(B,C)** Statistical analysis of protein bands. **(D)** Immunohistochemistry results (ZO-1 and Occludin). **(E,F)** Statistical analysis ZO-1 of IHC score. **(F)** Statistical analysis occludin of IHC score. The data are expressed as the means ± SDs (*n* = 5). *P < 0.05; **P < 0.01.

Both the NF-κB and MAPK pathways are important inflammatory signalling pathways. We further studied the effect of GML on p65, ERK and their phosphorylation levels by western blotting in the colon tissue. The p-p65 and p-MAPK levels of the DSS group were increased compared with those of the control group, but the p-p65 and p-MAPK levels of the GML group were significantly lower than those of the DSS group (Figures 3A–D), indicating that the activation of the NF-κB and MAPK signalling pathways was inhibited. To further investigate the effect of GML on intestinal tight junction proteins, we collected intestinal tissues for IHC analysis. Compared with the expression in the control group, the expression of ZO-1 and Occludin was decreased in the DSS group; however, the expression of ZO-1 and Occludin increased after GML treatment (Figure 3E). Negative control was established without primary antibody. The results showed that GML could alleviate DSS-induced damage and tight junction protein deficiency.

### GML could ameliorates DSS-induced acute colitis by inhibiting infiltration of Th17, neutrophils, macrophages

**Figure 4:**
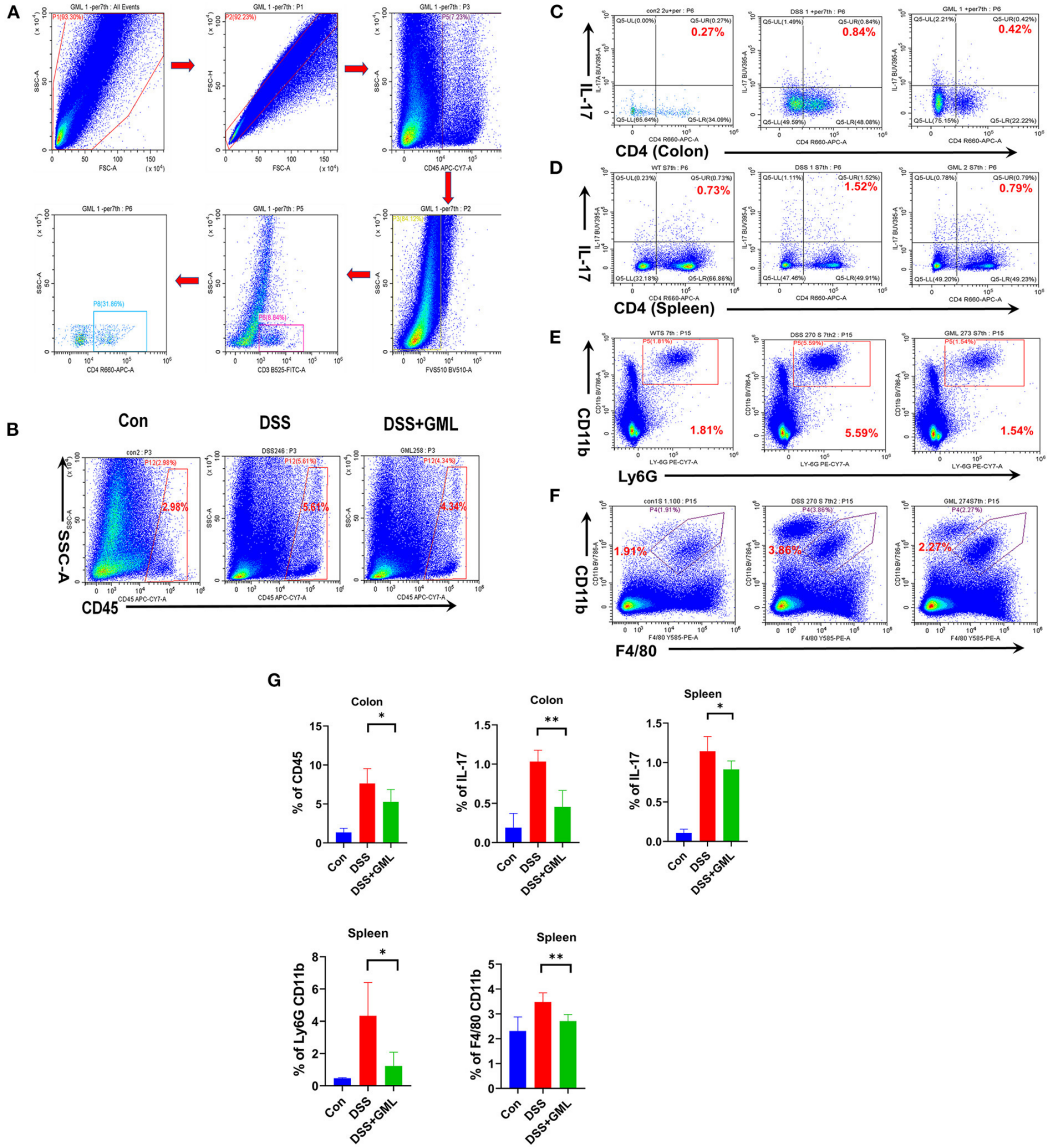
GML could ameliorates DSS-induced acute colitis by inhibiting infiltration of Th17, neutrophils, macrophages. **(A)** Gating strategy for flow cytometric analysis. **(B,C)** CD45 and Th17 cells from LPMCs were detected by flow cytometry. **(D–F)** Th17 cells, macrophages and neutrophils in the spleen were detected by flow cytometry. **(G)** The results were computed and graphed by graphPad. The data are expressed as the means ± SDs (*n* = 5). *P < 0.05; **P < 0.01.

Lymphocytes of the laminar propria of the colon were isolated and stained with Fixable Stain 510 and Fc-block. Then, the cells were stained for the cell markers CD45, CD3, CD4+, Ly-6G, CD11b, and IL-17A to study the effects of GML treatment on Th17, CD4+, neutrophils, and macrophages. Gating strategy for flow cytometric are shown in Figure 4A. Compared with the levels in the Con group, the CD45+ and IL-17A+ levels (% of IL17 on CD4 gate) in the LPMCs were significantly upregulated in the DSS group, CD4-positive and IL-17-positive (CD4+IL-17+) cells as Th17 cells. However, the application of GML significantly reversed this effect; the CD45+ and IL-17A+ levels were significantly decreased (Figures 4B,C). Compared with the Con group, the production of Th17 cells, neutrophils and macrophages in the spleen of the DSS group was significantly increased, and the production of Th17 cells, neutrophils and macrophages in the GML+DSS group was significantly decreased (Figures 4D–F). We also further explored the change of CD4 T cells between the seventh and the eleventh day. And we found that CD4 T cell was significantly decreased both at day 7 and day 11 (Supplementary Figure S3). These results suggest that GML could regulate the balance of the immune system.

### GML increases the abundance of akkermansia and lactobacillus

**Figure 5:**
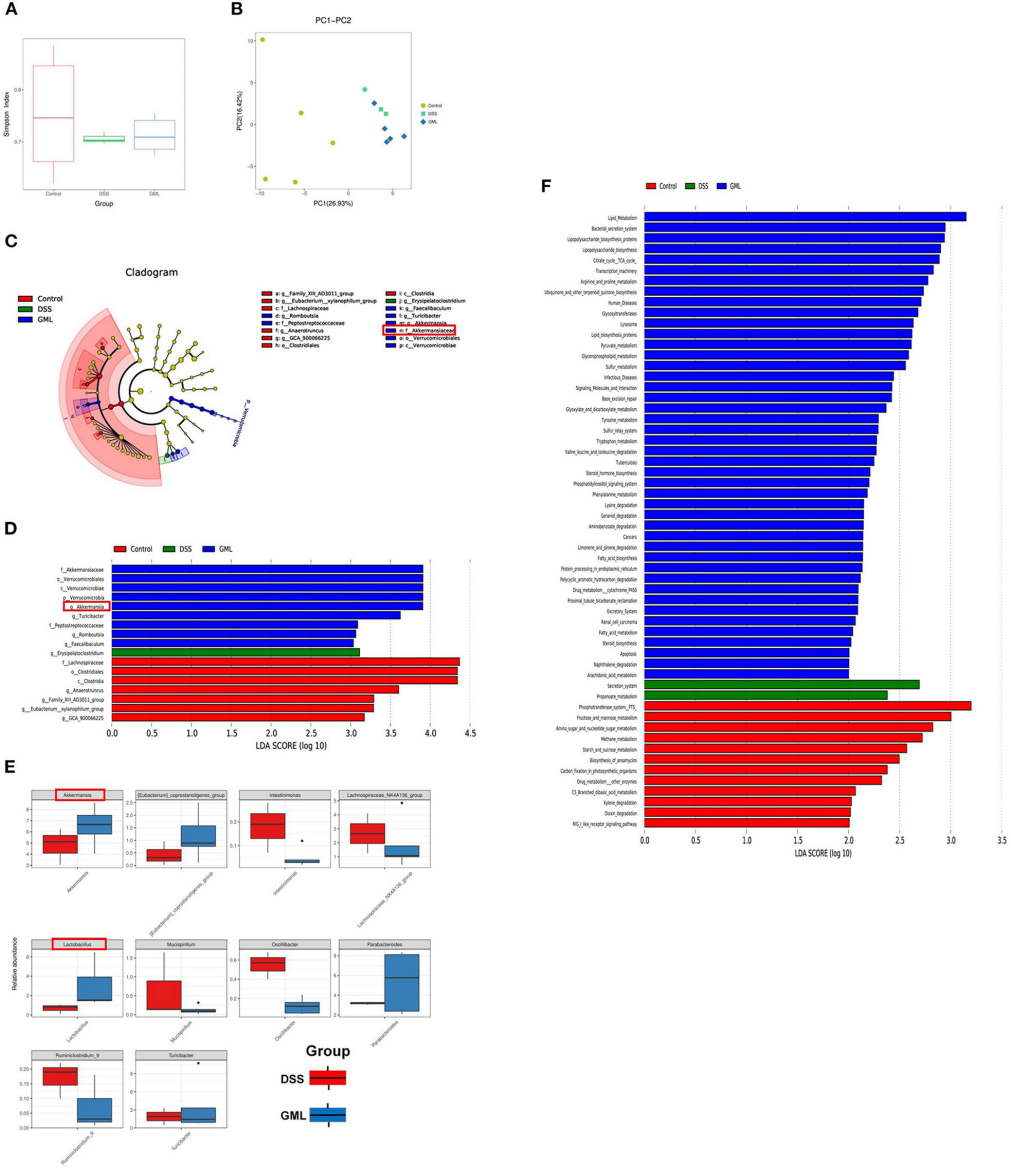
GML changes the community structure of the gut microbiota (*n* = 5). **(A)** Simpson's index. **(B)** Principal coordinate analysis (PCOA) of the caecal microbiota structure measured by weighted UniFrac distances. **(C)** Taxonomic cladogram of LEfSe analysis results. The circle from inside to outside indicates the rank from kingdom to species, and the circle size represents the taxon abundance in the community. **(D)** Linear discriminant analysis (LDA) score for different taxon abundances. **(E)** Results of the Metastats analysis. **(F)** Differentially abundant pathways of the microflora at various KEGG levels. The data are expressed as the means ± SDs (*n* = 5). *P < 0.05.

The 16S rDNA phylogenetic method was used to evaluate the colonic microbiomes of differently treated mice (97% similarity). The α diversity of the microbiome is indicated by Simpson's index (Figure 5A). PCA based on UniFrac distances was used to analyse the structural changes in the intestinal microbiota (Figure 5B). In addition, the changes in key bacteria in the species cluster evolutionary tree showed that Erysipelatoclostridium (pathogenic bacteria) played a key role in the DSS group, while Akkermansia, Turicibacter, Faecalibaculum, Peptostreptococcaceae, and Romboutsia played a key role in the DSS+GML group. Akkermansia, which has anti-inflammatory and anticancer effects, is a probiotic. Turicibacter can perform fermentation to produce lactic acid, and Faecalibaculum, Peptostreptococcaceae and Romboutsia can produce short-chain fatty acids (Figure 5C). Linear discriminant analysis (LDA) scores for the abundance levels of different taxa showed that DSS-induced colitis increased the abundance of pathogens such as Erysipelatoclostridium (43). After GML treatment, commensal bacterium such as Akkermansia, Turicibacter, Faecalibaculum, Peptostreptococcaceae and Romboutsia were significantly enriched (Figure 5D).

In addition, the results of the Metastats analysis showed that Intestinimonas, Lachnospiraceae_NK4A136_group, Mucispirillum, Ruminiclostridium_9, and Oscillibacter (a potentially harmful bacterium with pro-inflammatory effects) were significantly enriched after DSS treatment, while they were significantly depleted by the application of GML. Compared with their abundances in the DSS group, Akkermansia, Lactobacillus, Eubacterium, Coprostanoligenes_group, and Parabacteroides, which can regulate glucose and lipid metabolism, were significantly enriched after GML treatment (Figure 5E). Intestinal microecological community changes from the Phylum, Class, Order, Family, Genus, Species (Supplementary Figures S2A–F). These results suggest that GML can reduce the damage caused by DSS-induced colitis by increasing the abundances of probiotics. PICRUST software was used to predict the functions of microbial communities. KEGG pathway analysis showed that supplementation with GML significantly altered the functions of the microbial community compared with those in the DSS group (Figure 5F). The main pathways in the DSS group were secretion system and propanoate metabolism, while after GML treatment, the main pathways were lipid metabolism, bacterial secretion system and lipopolysaccharide biosynthesis proteins.

## Discussion

IBD is recognized as one of the challenging modern medical diseases. The recurrence of and prolonged failure to recover from can be physically and mentally distressing for patients, seriously affecting their quality of life and placing a heavy burden on the economy (3). Moreover, there are significant safety problems associated with the use of currently available drugs in long-term practical strategies due to the severe side effects (9). GML is a kind of medium-chain fatty acid that naturally occurs in breast milk (12). According to the literature, if GML is removed from breast milk, the ability of breast milk to eliminate harmful bacteria such as Staphylococcus is greatly reduced. In addition, GML also shows good anti-HIV and other antiviral effects (34). However, the specific mechanism of action of GML against harmful pathogens is poorly explored. Moreover, the effect of GML in alleviating colitis has not been reported.

To date, a colitis model induced by DSS has been widely used to study colitis and IBD. The changes in BW, colon length and spleen weight in mice are important indices for evaluating the DSS-induced colitis model. In this study, mice treated with DSS showed significant loss of BW, shortened colon length, and increased spleen weight, which indicated the successful establishment of a colitis model (44, 45). However, the mice regained BW faster, had longer intestines, and showed significantly reduced spleen weight after GML treatment, suggesting that GML could alleviate DSS-induced colitis. In addition, the results of HE staining showed that the pathological damage of DSS-induced colitis was alleviated in mice treated with GML. The lesions were characterized by reduced inflammatory cell infiltration and slight epithelial ulceration. The pathological structure was more complete than that of the DSS group.

Cytokines play an important role in the regulation of colitis (46–48). It has been reported that GML has a good effect on decreasing blood glucose and lipid levels in obese mice and reduces the production of inflammatory cytokines (TNF-α, LPS and IL-6) in mice (35, 36). We extracted total RNA from intestinal tissue for RT-qPCR, and the results showed that the levels of the pro-inflammatory factors TNF-α, IL-1β and IL-1α were decreased after GML treatment, while those of the inhibitory factors IL-10 and TGF-β were increased. Moreover, the ELISA results showed that the IL-1β level was decreased, while the TGF-β level was increased after GML treatment. In addition, the results of the cytometric bead array immunoassay analysis showed that the levels of the pro-inflammatory factors IFN-γ, TNF-α, and IL-6 were significantly increased after DSS treatment, while the application of GML reversed the change.

Impaired integrity of the intestinal barrier, which plays a key role in regulating intestinal permeability and the pathogenesis of colitis, is another cause of intestinal inflammation (49–51). It has been reported that GML can increase the expression of intestinal tight junction proteins such as ZO-1, Occludin and Claudin-1 in obese mice to protect the integrity of the intestinal barrier (35, 36). In this study, RT-qPCR, IHC and Western blot results showed that, compared with the expression in the control group, the expression of ZO-1 and Occludin in mice supplemented with DSS decreased sharply, while the expression of ZO-1 and Occludin increased after GML treatment. The results suggest that GML may ameliorate colitis by protecting the integrity of the intestinal barrier.

In addition, Th1, Th17 and Treg cells play an indispensable role in the occurrence and development of colitis. Proinflammatory factors secreted by Th1 and Th17 cells can induce the immune system to attack harmful pathogens and aggravate IBD to some extent (52–54). The results of the FCM analysis showed that GML could decrease the levels of CD4+ T and Th17 cells in LPMCs. Th17 cells in the spleen were also reduced after GML treatment. These results suggest that GML also has a good effect in regulating the immune balance.

Both the NF-κB and MAPK pathways, which play important regulatory roles in activating the production of inflammatory cytokines, are important inflammatory signalling pathways (55–57). The Western blot results showed that the application of GML reduced the expression of p-NF-κB and p-MAPK, thereby reducing the production of pro-inflammatory factors. These results suggest that these pathways are potential targets of GML in the treatment of intestinal inflammation and colitis.

In recent years, with the development of high-throughput sequencing of microflora, the intestinal microecology has attracted increasing attention. Some researchers believe that the development of IBD is closely related to the intestinal microecology (10, 58). Furthermore, it is noteworthy that changes in colon microbiota contribute to colitis pathogenesis (59, 60). Gut microbes were demonstrated to be an essential factor in intestinal inflammation in IBD (61). Some studies suggest that dysbiosis occur in IBD, and a broad microbial alteration pattern was revealed including reduction in diversity, decreased abundances of bacterial taxa within the Phyla Firmicutes and Bacteroides, and increases in the Gammaproteobacteria (62). Studies shown that A. parvulum causes colitis in a susceptible mouse model and that the host microbiota is required for colitis development (63). Based on this hypothesis, clinical treatments have been developed to improve the condition of IBD patients by transplanting faecal bacteria from healthy people (64–67). Moreover, probiotics have been reported to improve DSS-induced colitis (68–71). 16S rDNA sequencing analysis showed that GML could increase the composition and diversity of the intestinal microbial community. The results of the species cluster evolutionary tree showed that Erysipelatoclostridium played a key role in the DSS group, while Akkermansia, Turicibacter, Faecalibaculum, Peptostreptococcaceae, and Romboutsia played a key role in the DSS+GML group. Akkermansia has anti-inflammatory and anticancer effects (72–74). In addition, the results of the Metastats analysis showed that GML could increase the abundances of Akkermansia and Lactobacillus compared with the abundances in the DSS group. Moreover, PICRUST software was used to predict the functions of the microbial community, and the results showed that the lipid metabolism pathway was significantly different after GML treatment. These results suggest that GML may ameliorate colitis by enriching probiotics.

In conclusion, these results indicate that oral administration of GML could slow down the occurrence of DSS-induced colitis by regulating the immune balance, protecting the intestinal mucosal barrier, and improving the intestinal microecology. This study provides new insights into the biological function and therapeutic potential of GML in the treatment of IBD.

## Data availability statement

The data presented in the study are deposited in the NCBI SRA BioProject repository, accession number PRJNA865147.

## Ethics statement

The animal study was reviewed and approved by Animal Research Committee of Xiamen University.

## Author contributions

K-JH performed the study and drafted the manuscript. Y-NH, J-HD, and X-MO conducted RNA extraction and qRT-PCR assays. YLin, GG, and X-SC carried out Western blot assays. K-JH, YLi, and JL developed animal model and performed experiments, and K-JH analyzed the data. BG and J-LR revised the manuscript and supervised the whole experiment. All authors have read and approved the final submitted manuscript.

## Funding

This study was supported by the National Natural Science Foundation of China (Nos. 81970460 and 81770548) in China, and the Key Laboratory of Intestinal Microbiome and Human Health in Xiamen, China.

## Conflict of interest

The authors declare that the research was conducted in the absence of any commercial or financial relationships that could be construed as a potential conflict of interest.

## Publisher's note

All claims expressed in this article are solely those of the authors and do not necessarily represent those of their affiliated organizations, or those of the publisher, the editors and the reviewers. Any product that may be evaluated in this article, or claim that may be made by its manufacturer, is not guaranteed or endorsed by the publisher.

## Supplementary material

Supplementary Figure S1 **(A)** The body weight of the mice was expressed as a percentage change relative to the initial body weight. **(B)** The spleen weight was measured. **(C)** The standard curve of the inflammatory cytokines.

Supplementary Figure S2 **(A–F)** Intestinal microecological community changes from the Phylum, Class, Order, Family, Genus, Species.

Supplementary Figure S3 Changes of CD4 T cells between the seventh and the eleventh day (*n* = 3). The data are expressed as the means ± SDs (*n* = 3). ^*^*P* < 0.05; ^**^*P* < 0.01.

Supplementary Table 1 Primer sequences.
