# Supplementary figures and images for: Glycerol monolaurate ameliorates DSS-induced acute colitis by inhibiting infiltration of Th17, neutrophils, macrophages and altering the gut microbiota
Source: Front Nutr. 2022 Aug 12;9:911315. doi: 10.3389/fnut.2022.911315 (PMC9413164; doi:10.3389/fnut.2022.911315)

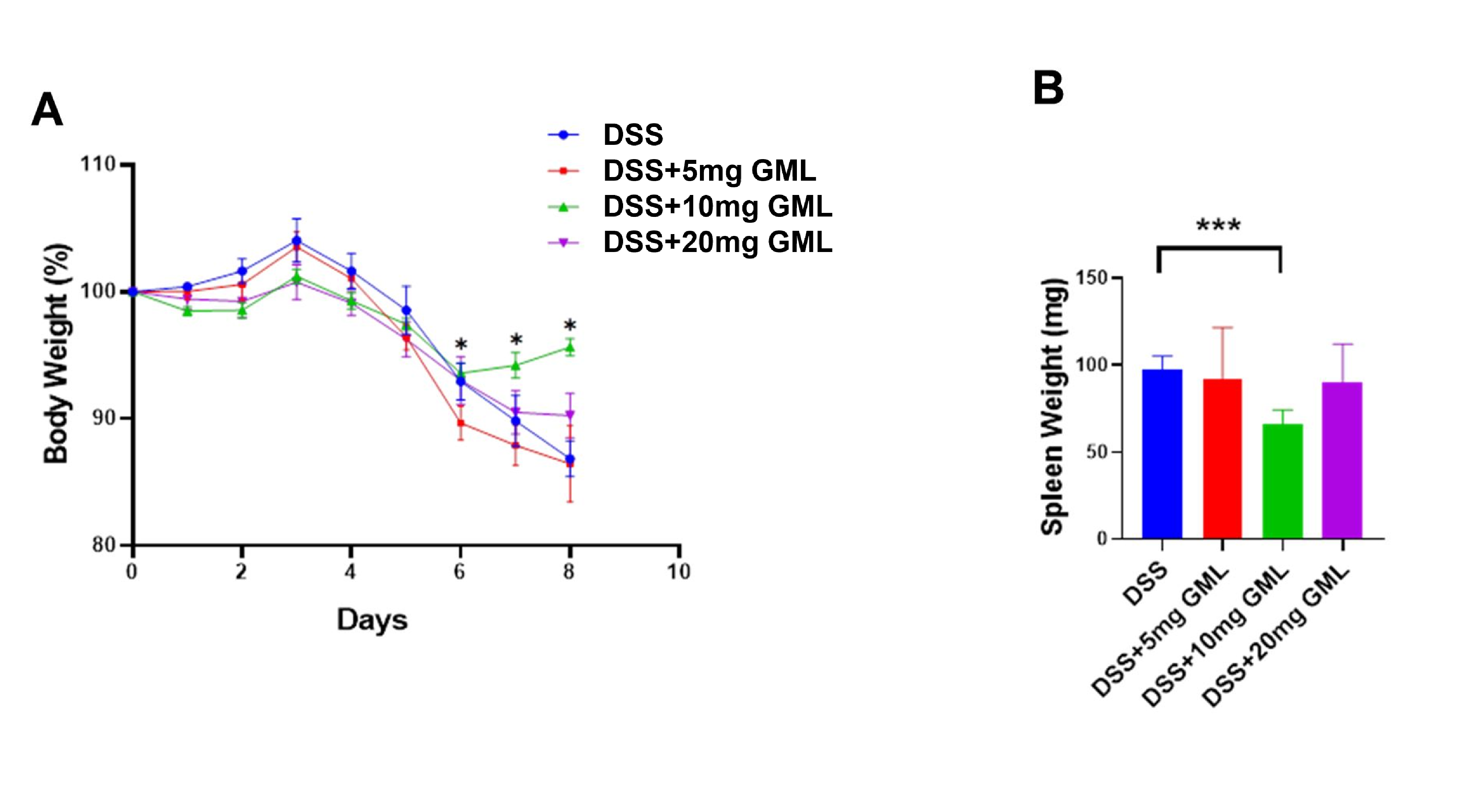

Supplement: Supplementary Figure S1 — (A) The body weight of the mice was expressed as a percentage change relative to the initial body weight. (B) The spleen weight was measured. (C) The standard curve of the inflammatory cytokines. [file Data_Sheet_1.zip › Supplementary Figures/Figure 1A,B.TIFF]

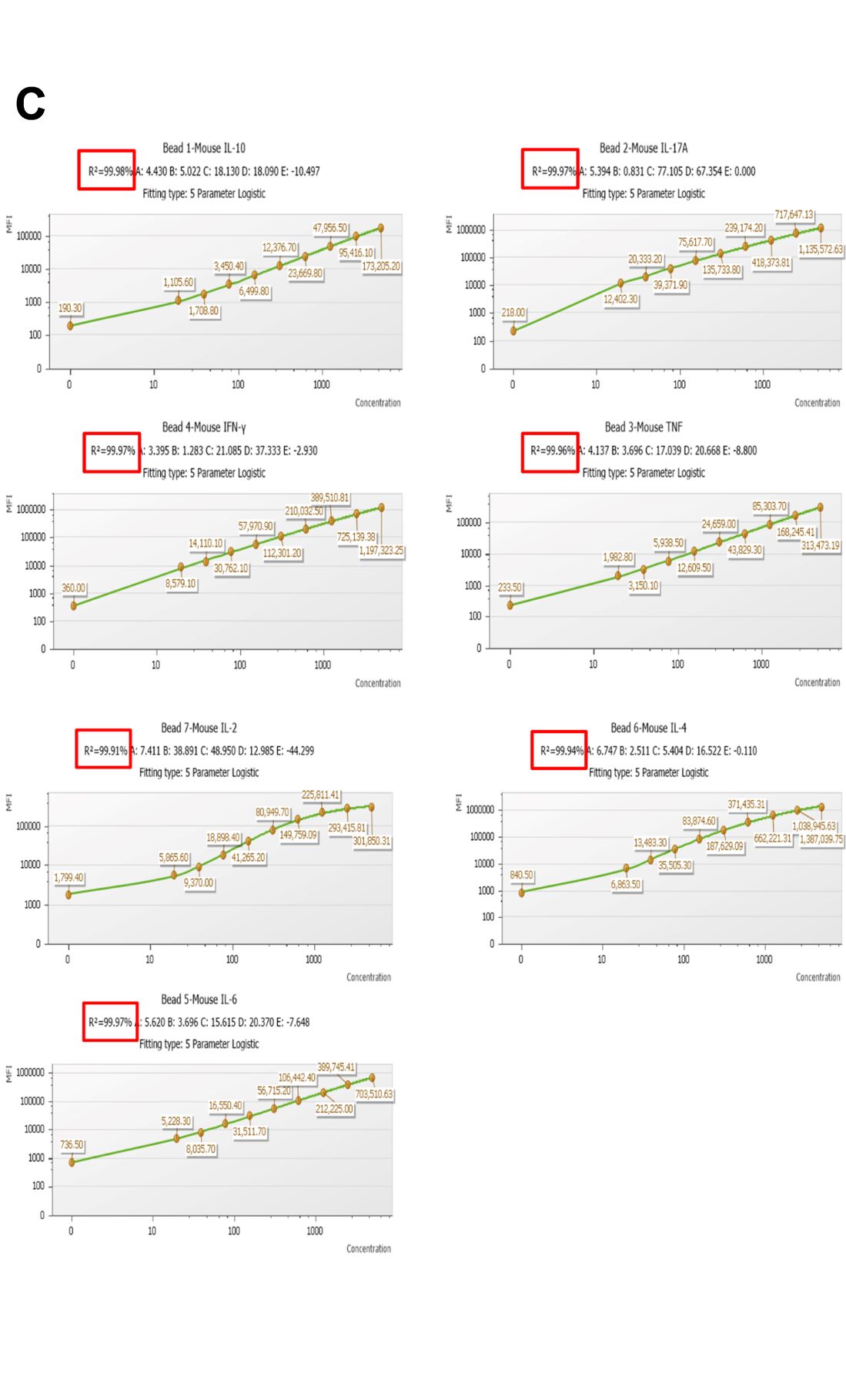

Supplement: Supplementary Figure S1 — (A) The body weight of the mice was expressed as a percentage change relative to the initial body weight. (B) The spleen weight was measured. (C) The standard curve of the inflammatory cytokines. [file Data_Sheet_1.zip › Supplementary Figures/Figure S1C.TIF]

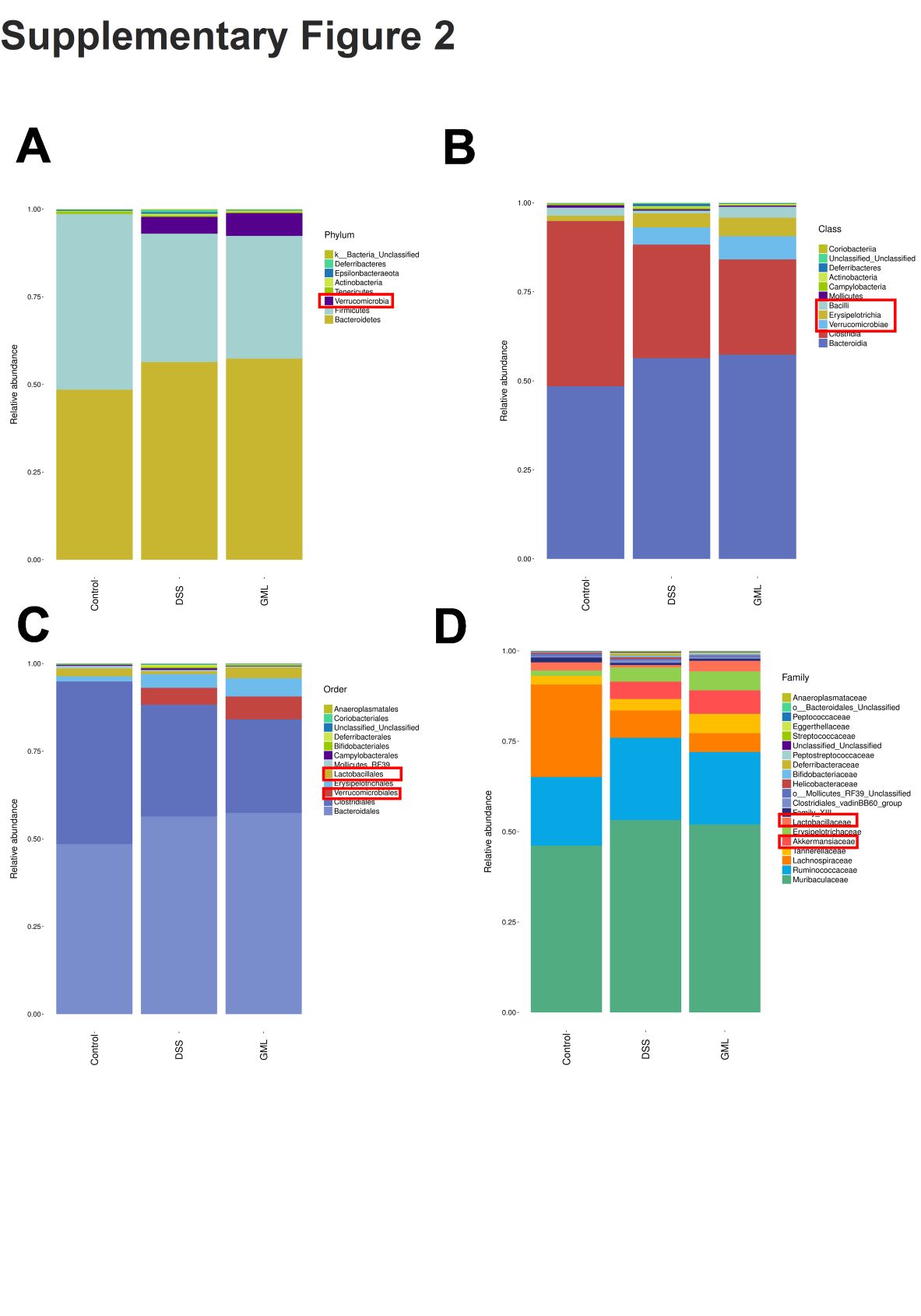

Supplement: Supplementary Figure S1 — (A) The body weight of the mice was expressed as a percentage change relative to the initial body weight. (B) The spleen weight was measured. (C) The standard curve of the inflammatory cytokines. [file Data_Sheet_1.zip › Supplementary Figures/Figure S2A-D.TIF]

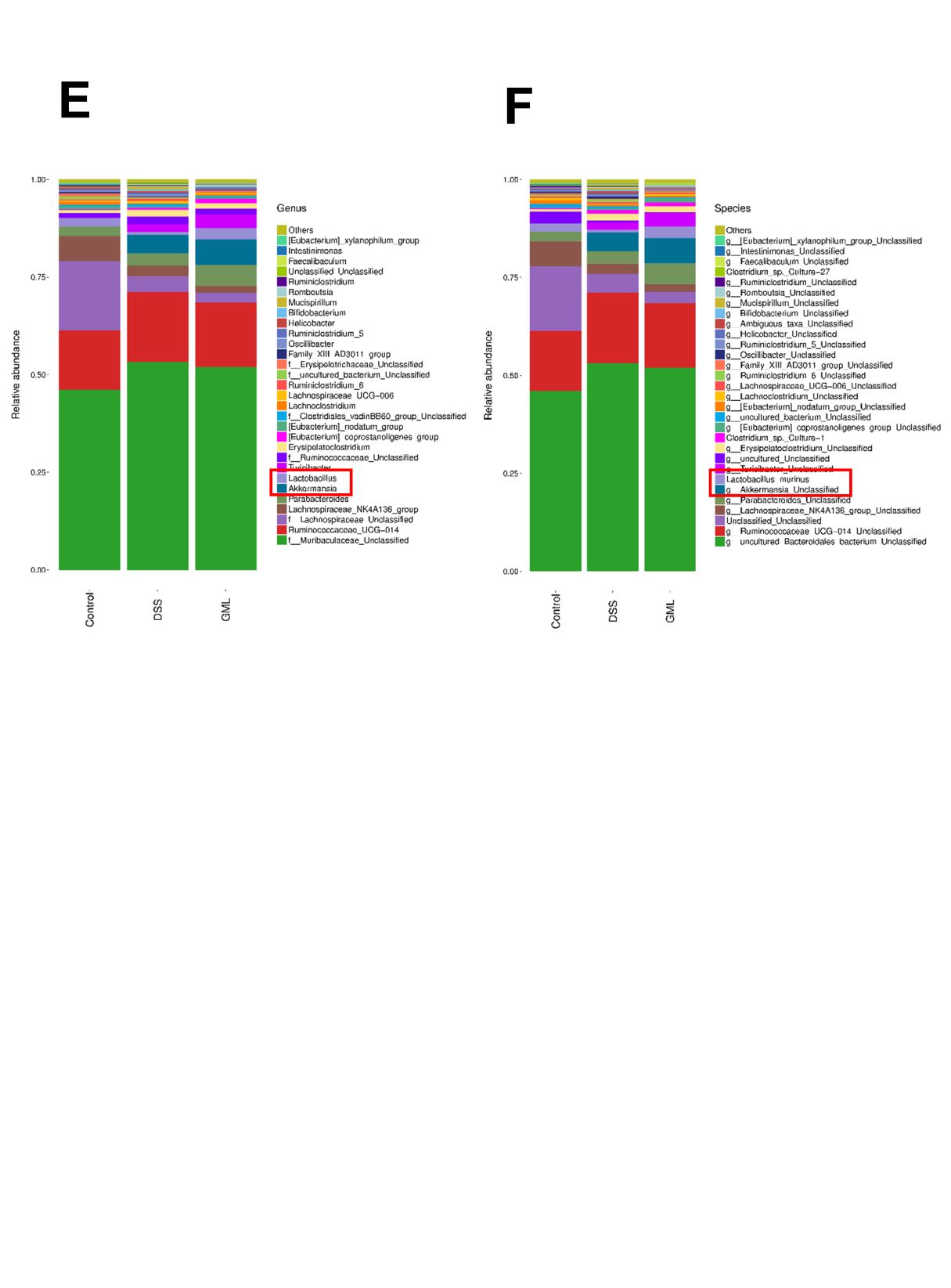

Supplement: Supplementary Figure S1 — (A) The body weight of the mice was expressed as a percentage change relative to the initial body weight. (B) The spleen weight was measured. (C) The standard curve of the inflammatory cytokines. [file Data_Sheet_1.zip › Supplementary Figures/Figure S2E,F.TIF]

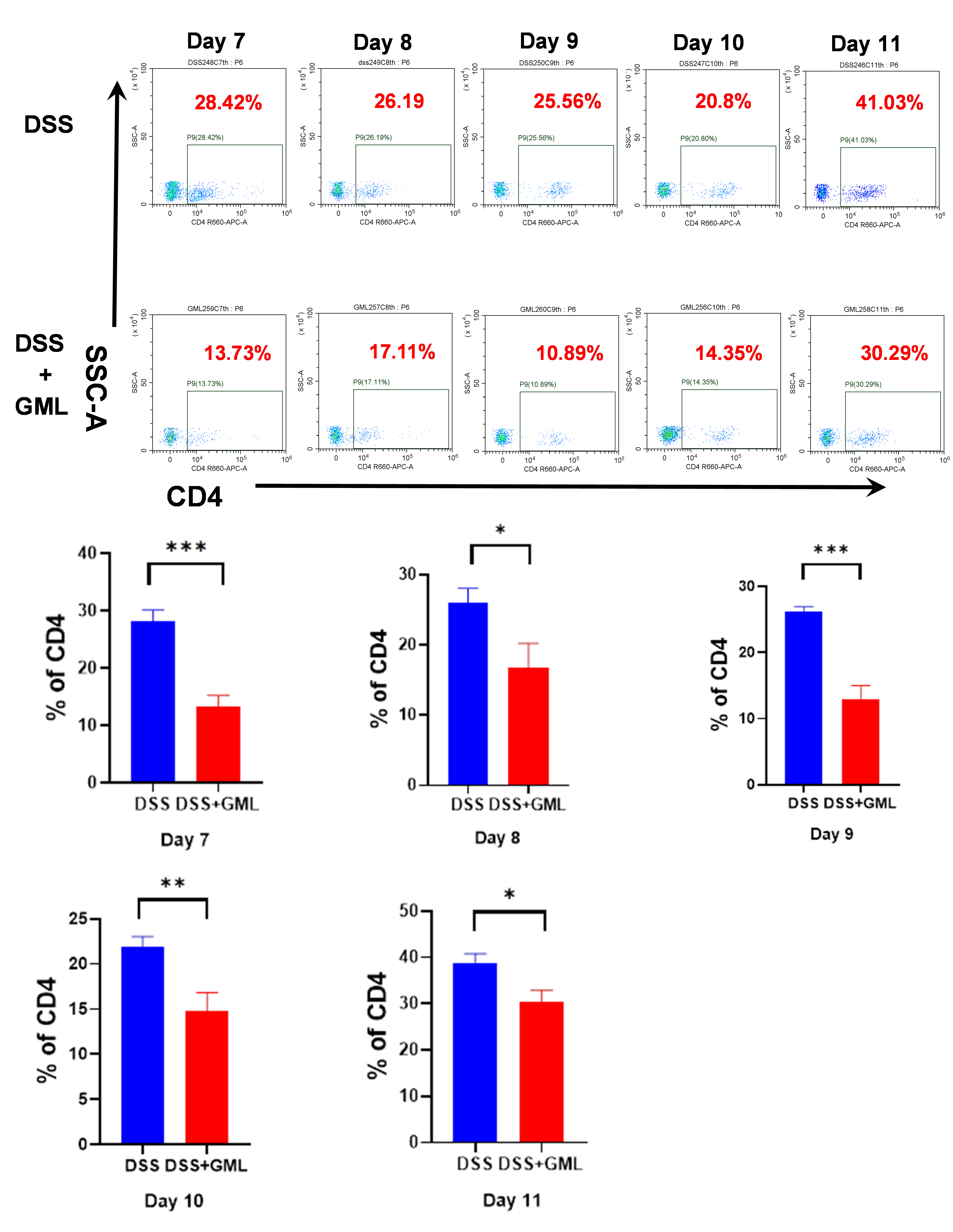

Supplement: Supplementary Figure S1 — (A) The body weight of the mice was expressed as a percentage change relative to the initial body weight. (B) The spleen weight was measured. (C) The standard curve of the inflammatory cytokines. [file Data_Sheet_1.zip › Supplementary Figures/Figure S3.TIF]
