# Supplementary material for: Glycerol monolaurate ameliorates DSS-induced acute colitis by inhibiting infiltration of Th17, neutrophils, macrophages and altering the gut microbiota
Source: Front Nutr. 2022 Aug 12;9:911315. doi: 10.3389/fnut.2022.911315 (PMC9413164; doi:10.3389/fnut.2022.911315)
Supplement: Supplementary Table 1 — Primer sequences. [file Table_1.DOCX]

**Table 1** Primer sequences

| *** Primer** | ***Primer sequences (5'--3')** |
| --- | --- |
| TNF-α Forward | CCTGTAGCCCACGTCGTAG |
| TNF-α Reverse | GGGAGTAGACAAGGTACAACCC |
| IL-1α Forward | ACGGCTGAGTTTCAGTGAGACC |
| IL-1α Reverse | CACTCTGGTAGGTGTAAGGTGC |
| IL-1β Forward | TGGACCTTCCAGGATGAGGACA |
| IL-1β Reverse | GTTCATCTCGGAGCCTGTAGTG |
| TGF-β Forward | TGACGTCACTGGAGTTGTACGG |
| TGF-β Reverse | GGTTCATGTCATGGATGGTGC |
| IL-10 Forward | GGTTGCCAAGCCTTATCGGA |
| IL-10 Reverse | ACCTGCTCCACTGCCTTGCT |
| ZO-1 Forward | GCAAAGAGATGAGCGGGCTACC |
| ZO-1 Reverse | GTCATGCGAGCGACCTGAATGG |
| GAPDH Forward | TGTGTCCGTCGTGGATCTGA |
| GAPDH Reverse | TTGCTGTTGAAGTCGCAGGAG |

16s V3-V4 Forward CCTACGGRRBGCASCAGKVRVGAAT

16s V3-V4 Reverse GGACTACNVGGGTWTCTAATCC
